# Hormonal Contraception and Massive Pulmonary Embolism in a COVID-19 Ambulatory Patient: A Case Report

**DOI:** 10.3390/clinpract11040105

**Published:** 2021-11-26

**Authors:** Laura Valenzuela-Vallejo, David Corredor-Orlandelli, Sergio Alzate-Ricaurte, Valentina Hernández-Santamaría, Juan Felipe Aguirre-Ruiz, Adwar Peña-Peña

**Affiliations:** 1 School of Medicine and Health Sciences, Universidad del Rosario, Bogotá 110111, Colombia; david.corredor01@urosario.edu.co (D.C.-O.); sergio.alzater@urosario.edu.co (S.A.-R.); Valentina.hernandezs@urosario.edu.co (V.H.-S.); 2 Internal Medicine Department Fundación Cardioinfantil—LaCardio, Bogotá 110111, Colombia; juanf.aguirre@urosario.edu.co (J.F.A.-R.); adwar_1@hotmail.com (A.P.-P.)

**Keywords:** COVID-19, pulmonary embolism, thrombosis, oral contraception, massive embolism

## Abstract

Coronavirus 19 disease (COVID-19) presents a highly variable clinical presentation and course, ranging from asymptomatic patients to rapidly progressive, fatal pneumonia. The known heterogeneous outcomes can affect both previously healthy patients and those with significant comorbidities, who develop clinical courses with possibly more multisystemic compromise. Likewise, the development of thrombotic phenomena during the acute course of the disease is associated with complications that worsen patient prognosis. We present a case report of a 45-year-old multiparous patient with a history of overweight and chronic use of oral hormonal contraception with low doses of levonorgestrel and estradiol as the only risk factors favoring the development of thrombotic events. During her outpatient COVID-19 clinical course, she developed massive pulmonary thromboembolism resulting in secondary obstructive shock, which required pharmacological thrombolysis. At discharge, hormonal contraception was considered contraindicated, and the patient was released from our institution with continued oral anticoagulant therapy. COVID-19 infection, contraceptive hormone therapy, and overweight are known risk factors for the development of thromboembolic events. The impact of their concomitance has not been studied to date. From our experience, we discuss the impact these risk factors have when present together and invite others to report similar cases.

## 1. Introduction

Severe acute respiratory syndrome coronavirus 2 (SARS-CoV-2) infection presents a broad and variable clinical picture ranging from asymptomatic patients to life-threatening acute respiratory distress syndrome [1]. Patients with COVID-19 disease frequently experience a hypercoagulable state that increases the development of thrombotic events and worse clinical outcomes, associated with an increased inflammatory response, prolonged immobilization, and transient hypoxia secondary to pulmonary compromise. A higher prevalence of deep venous thrombosis (DVT), which can progress to pulmonary thromboembolism (PTE), has been found mainly in critically ill patients with prolonged stays in the intensive care unit (ICU). However, literature that addresses the concomitance with other risk factors like oral hormonal contraceptives (OHP) is lacking [2].

A multicenter study in France, which excluded ICU patients, reported an incidence of 8.3% in 1240 patients [3]. In Italy, an incidence of 14% in 224 patients admitted in seven hospitals was reported [4]. Nevertheless, typical risk factors for PTE have been widely described, such as obesity, previous thrombotic event, history of malignancy, smoking, increased age or frailty, and chronic pathologies [5]. Venous thromboembolism (VTE) phenomena occur as a relatively common complication in SARS-CoV-2 infection, although literature addressing the concomitance with other risk factors such as the use of oral hormonal contraceptives (OHPs) is lacking.

Hormonal contraceptive methods are widely used and are associated with increased thrombotic events, mainly in estrogenic-containing therapies such as ethinyl estradiol. On the other hand, hormonal contraceptive components such as progestins have not shown an increase in thrombotic risk so far [6]. The hypercoagulable state found in OHP users results from changes in procoagulant factors and an altered fibrinolytic balance. The annual incidence of VTE in OHP users is 1.17 per 10,000 persons, with a known increase in recent years. This is thought to be due to the rise in the number of OHP prescriptions over time [7].

To date, clinically significant thrombotic complications (such as pulmonary embolism and bilateral popliteal vein thrombosis) have been reported in mild SARS-CoV-2 infections [8,9,10]. However, only one case report described this kind of event in the context of pre-existing chronic use of OHP [11]. We present another clinical case in which known prothrombotic phenomena, such as overweight, chronic OHP use, and COVID-19, converge resulting in massive PTE, raising the possibility of a possible additive effect when facing the aforementioned risk factors.

## 2. Case Description

### 2.1. Emergency Room Consultation and COVID-19 Diagnosis

A 45-year-old, multiparous, overweight female with a history of OHP use for 13 years (levonorgestrel 0.15 mg and estradiol 0.03 mg daily) consulted the emergency room of our institution following a one-week clinical course of worsening dyspnea, general malaise, headache, and ageusia. At admission, the patient reported dyspnea at rest, associated with intermittent retrosternal oppressive chest pain radiating to the back. The physical exam revealed pulmonary aggregates on auscultation, and her vital signs showed tachypnea, tachycardia, and desaturation. Oxygen therapy was started, requiring a non-rebreathing mask at 12 L/min to maintain adequate oxygen saturation. RT-PCR test for SARS-CoV-2 was indicated. Arterial blood gases analysis showed a PAO_2_/FIO_2_ ratio of 56, and the patient was then transferred to the respiratory intensive care unit (ICU).

### 2.2. Massive Pulmonary Embolism and Initial ICU Stay

Her COVID-19 diagnosis was confirmed with the positive results of the RT-PCR test for SARS-CoV-2 (50 copies of RNA/reaction). Laboratory test results showed positive severity predictors, including an elevation of D-dimer (>20 mg/L), troponin I (0.150 ng/mL), ferritin (2934 ng/mL), and lactate dehydrogenase (879 U/L) levels. Other admission paraclinical tests showed leukocytosis, neutrophilia, lymphopenia, mild thrombocytopenia, and elevation of transaminases more than three times the laboratory upper limit. Because of the risk of bacterial pneumonia co-infection, ampicillin-sulbactam was started as empiric antibiotic treatment.

Due to significant elevation of the D-dimer, a CT pulmonary angiography (CTPA) was taken according to the YEARS protocol. The results of the CTPA showed a massive PTE with compromise to the posterior basal segmental artery of the left lower lobe, inferior lingula, and apical-posterior segment of the left upper lobe. An echocardiogram was performed, showing right ventricular dysfunction. Systemic thrombolysis with r-tPA (alteplase) 100 mg infusion over 2 h was administered according to the European Society of Cardiology guidelines.

### 2.3. ICU-Related Complications

After treatment, the patient showed an improvement in her hemodynamic and ventilatory patterns. Nonetheless, during the hospital stay, the patient displayed additional complications: (1) septic shock secondary to a hospital-acquired infection requiring broad-spectrum antibiotics and hemodynamic support therapy, (2) respiratory failure with invasive mechanical ventilation support requirement and subsequent tracheostomy, and (3) severe anemia with the need for blood transfusions.

### 2.4. Discharge and Outpatient Plan

**Figure 1:**
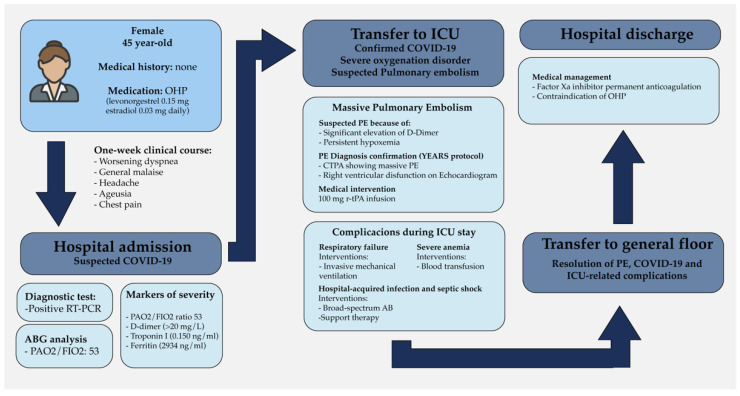
Timeline of events of the clinical case showing laboratory results. Clinical findings and medical decisions. OHP = oral hormonal contraceptives, RT-PCR = retro-transcriptase polymerase chain reaction, ICU = intensive care unit, PE= pulmonary embolism, AB = antibiotics.

The patient had an adequate response to treatment with satisfactory clinical evolution, successful extubation, and transfer to the general hospital floor. The patient was evaluated by the OB-GYN attending physician, who contraindicated the further use of estrogenic hormonal contraceptives. Finally, the patient was discharged from our institution with indefinite anticoagulation therapy with a factor-XA inhibitor. The entire hospital stay course is represented in Figure 1.

## 3. Discussion

Host response to SARS-CoV-2 infection is difficult to predict. The clinical course of disease does not always correlate directly with known risk factors identified to date. Venous thromboembolism is a multifactorial disease for which numerous risk factors, such as COVID-19 have been identified to increase the risk of developing thrombotic phenomena. Venous thromboembolism in COVID-19 has been more frequently observed in patients requiring ICU stays and patients with high scores on the thrombosis prediction scale [12]. However, identifying the risk in patients with non-severe COVID-19 or the outpatient setting is challenging.

Paraclinical markers are widely used in patients’ workup for the detection of thrombotic events. D-dimer, a fibrin degradation product, is frequently used to rule out VTE. However, it is elevated in COVID-19 in up to 61.7% of patients, even in the absence of PTE and DVT [13]. Similarly, an increase has been reported to be positively correlated with age, length of hospital stay, non-thrombotic pulmonary compromise, neutrophil count, and inversely related to lymphocyte count. The usefulness of D-dimer as a diagnostic aid in patients with suspected PTE in COVID-19 disease is limited. The cutoff point values described for patients without a diagnosis of COVID-19 (500 µg/L or adjusted for age) cannot be extrapolated to the COVID-19 patient. Different cutoff values of 1743 µg/L and 1371 µg/L have been proposed [4].

Prolonged immobilization presents a prothrombotic risk factor; however, knowing the degree of immobilization in the outpatient setting is challenging. In the medical literature, multiple authors have suggested starting thromboprophylaxis in those with significant prothrombotic risk factors and low risk of bleeding, such as malignancy or previous DVT. For instance, guidelines such as ASH and CHEST suggest using prophylactic-intensity anticoagulation with LMWH for COVID-19 admitted patients [14,15]. On the other hand, some recommendations known for DVT prophylactic strategies such as frequent mobilization could be effective in patients with mild COVID-19 clinical courses who can functionally perform daily activities. Discontinuing prothrombotic drugs such as OHPs, selective cyclooxygenase-2 (COX-2) inhibitors, or vitamin K/E supplementation has to be considered [16]. Recent society guidelines have suggested initiation of thromboprophylaxis in the outpatient setting based on results of risk stratification scales, with further ongoing clinical trial results needed for definite recommendations [17].

Regarding outpatient management of thromboprophylaxis, data has suggested the benefits and importance of evaluating out-of-hospital risk, emphasizing early detection of worsening dyspnea or clinical signs of DVT. Other studies emphasize the importance of telemedicine follow-ups, allowing physicians to actively search for patients who are possibly suffering from DVT, even suggesting the need for D-dimer testing as a follow-up tool for patients discharged from hospitals. However, the use of risk stratification through laboratory testing has not been studied in outpatients as a prognostic prothrombotic tool.

Furthermore, the wide interindividual variability of SARS-CoV-2 infection is evident. The fact that our patient progressed to a severe disease phenotype without any associated comorbidity reinforces the importance of differentiating individual characteristics of each patient’s immunologic and genetic substrate [18]. Further population-specific studies are needed to elucidate this phenomenon.

Although OHP increases the risk of thrombosis by 1.5–2 fold, resulting in 14–20 thrombotic events per 10,000 women [19], there is scarce evidence of its additive risk effect on acute, subacute, or chronic SARS-CoV-2 infection. A limitation for this case was the absence of a complete evaluation of additional procoagulant risk factors and the differentiation on which factor primarily contributed to the patients’ clinical outcome. Nevertheless, the patient has no personal or familiar thrombotic history. This article is the second case report of massive PTE in COVID-19 disease without associated risk factors other than chronic OHP use, suggesting a possible relationship and elucidating the clinical challenges posed by the disease.
